# The potential adverse effects of hypodermic glucagon‐like peptide ‐1 receptor agonist on patients with type 2 diabetes: A population‐based study

**DOI:** 10.1111/1753-0407.70013

**Published:** 2024-10-22

**Authors:** Zhiyuan Cheng, Shuang Wang, Fu‐rong Li, Cheng Jin, Chunbao Mo, Jing Zheng, Xia Li, Fengchao Liang, Jinkui Yang, Dongfeng Gu

**Affiliations:** ^1^ School of Public Health and Emergency Management Southern University of Science and Technology Shenzhen China; ^2^ Shenzhen Key Laboratory of Cardiovascular Health and Precision Medicine Southern University of Science and Technology Shenzhen China; ^3^ Shenzhen Health Development Research and Data Management Center Shenzhen China; ^4^ School of Medicine Southern University of Science and Technology Shenzhen China; ^5^ Department of Endocrinology, Beijing Tongren Hospital Capital Medical University Beijing China

**Keywords:** clinical adverse outcomes, GLP‐1 receptor agonists, type 2 diabetes

## Abstract

**Background:**

Glucagon‐like peptide‐1 receptor agonists (GLP‐1 RAs), a class of injectable antidiabetic drugs, have shown significant efficacies in improving glycemic and weight control in patients with type 2 diabetes (T2D). However, the long‐term safety of GLP‐1 RAs remains insufficiently studied. This study aimed to provide real‐world evidence on potential adverse outcomes associated with GLP‐1 RAs use in T2D patients without major chronic diseases including impaired cardiac or renal function.

**Methods:**

We conducted a retrospective cohort study involving 7746 T2D patients on GLP‐1 RAs in Shenzhen, China. They were compared with 124 371 metformin‐only users and 36 146 insulin‐only users, forming two therapy control groups. GLP‐1 RAs users were also further 1:2 paired with the control groups. Competing risk survival analyses were conducted to assess the incidence risks, presenting subdistributional hazard ratios (sHRs) with 95% confidence intervals (CIs) for various adverse outcomes associated with GLP‐1 RAs use.

**Results:**

Compared with metformin‐only users, GLP‐1 RAs use was associated with increased risks of various adverse outcomes (sHRs with 95% CIs), including pancreatitis (2.01, 1.24–3.24), acute nephritis (3.20, 2.17–4.70), kidney failure (3.73, 2.74–5.08), thyroid cancer (2.25, 1.23–4.10), and thyroid dysfunction (1.27, 1.00–1.63), respectively; Similar results were also found when compared with insulin‐only users. Importantly, long‐term (≥12 months) GLP‐1 RAs use may further elevate the incidence risks of pancreatitis, acute nephritis, thyroid cancer, and thyroid dysfunction.

**Conclusion:**

Compared with traditional T2D treatments, GLP‐1 RAs use may be associated with increased risks of various adverse outcomes in a Chinese population. Cautions were strongly warranted in the use of GLP‐1 RAs. Further validation is crucial across diverse populations.

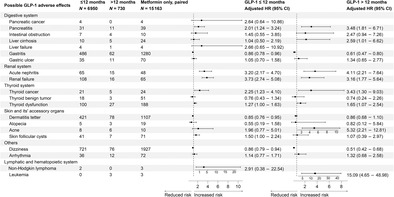

## Graphical abstract

Highlights

- GLP‐1 RAs may increase the incidence risks of various adverse outcomes.
- Prolonged use (over 12 months) of GLP‐1 RAs may further elevate these risks.
- Cautions were strongly recommended in clinical practice with risk–benefit balance, particularly for risks of pancreatitis, acute nephritis, kidney failure, thyroid cancer/dysfunction.

## 1 INTRODUCTION

The advent of glucagon‐like peptide‐1 receptor agonists (GLP‐1 RAs) has revolutionized the management of type 2 diabetes (T2D), offering not only improved glycemic control and a beneficial decrease in cardiovascular disease (CVD) risks but also a favorable effect on significant weight loss.^1^, ^2^, ^3^ Despite their increasing popularity, the safety profile of these drugs, particularly in real‐world settings, remains a subject of ongoing investigation.^4^, ^5^ Indeed, recent studies have raised concerns about potential adverse outcomes associated with GLP‐1 RAs use,^6^, ^7^ such as gastrointestinal disturbances, thyroid dysfunction, and acute organ injury.^8^, ^9^ However, the evidence is still insufficient to establish a clear association. This uncertainty may cause anxiety among patients and healthcare providers alike, potentially impacting treatment decisions and adherence to clinical treatments.

Establishing a comprehensive safety profile for GLP‐1 RAs is paramount, particularly given that the existing clinical trials may underrepresent key patient demographics and provide limited outcomes.^7^ These limitations compromised the generalizability of trial results to real‐world clinical scenarios, thereby accentuating the need for large‐scale, population‐based, real‐world studies to comprehensively examine the short‐term and long‐term safety of GLP‐1 RAs.^8^, ^10^ Moreover, the rapid approval and widespread adoption of GLP‐1 RAs by the drug & medicine regulatory agencies across multiple countries have further heightened the urgency to understand these drugs' safety profiles.

Given these multifaceted considerations, our study aimed to address the critical gap by investigating the potential adverse effects associated with GLP‐1 RAs use in a real‐world setting in a Chinese population. The results may offer invaluable insights into the risk–benefit balance of GLP‐1 RAs in clinical practice, thereby informing clinical decision‐making.^9^, ^11^

## 2 METHODS

### 2.1 Study population

This study is a clinical data‐based real‐world with retrospective cohort study design. Data of this study population originated from the Health Information Database of Shenzhen city in China, from the date of the first medical record included on January 1, 2016 to the last database update on September 1, 2022. Our database collected and centralized citizens' demographic data and covered all available medical information from registered health institutions in Shenzhen.

**FIGURE 1:**
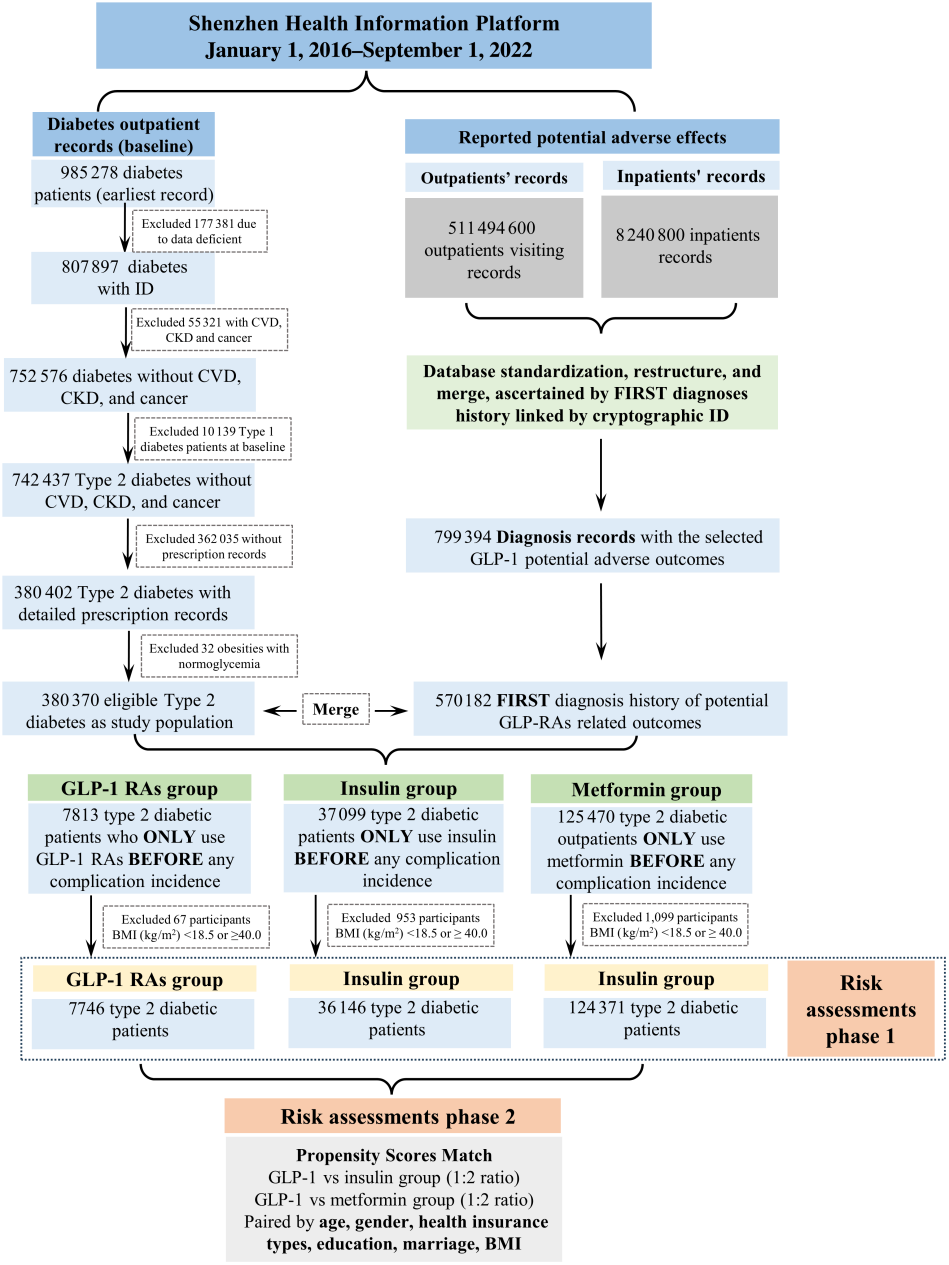
Flow‐chart of the excluding criteria. BMI, body mass index; CKD, chronic kidney disease; CVD, cardiovascular disease; GLP‐1 RA, glucagon‐like peptide‐1 receptor agonists.

As shown in Figure 1, a total of 985 278 diabetes outpatients were initially selected as the target population. Subsequently, 177 381 outpatients with missing IDs were excluded. We further excluded 55 321 outpatients who were diagnosed as prevalent status of CVD, chronic kidney disease, or cancer, along with 10 139 type 1 diabetes patients at baseline. Additionally, 362 035 outpatients were further excluded due to missing prescription data. To eliminate the potential confounding from GLP‐1 RAs use only for weight loss, we further excluded individuals who were obese with normoglycemia at baseline (body mass index [BMI] >28.0 kg/m^2^ and glycated hemoglobin A1c [HbA1c] <6.5% or fasting blood glucose <7.0 mmol/L, *n* = 32). This resulted in a total of 380 370 eligible T2D outpatients as the baseline population.

After the ascertainment of baseline data with an additional exclusion of T2D participants with extremum BMI values (BMI < 18.5 or BMI ≥ 40 kg/m^2^). The final analytic sample was restricted into 3 groups, comprising a total of 7746 T2D patients who were exclusively prescribed GLP‐1 RAs. The GLP‐1 RAs group was defined as any T2D patients who mainly prescribed GLP‐1 RAs lasting from the baseline date to the last follow‐up while the accumulative prescription period of any other antidiabetic medication <6 months. Additionally, this study integrated two control groups of T2D patients, namely those mainly receiving insulin therapy and those mainly receiving metformin therapy, representing distinct first‐line treatments in clinical practice. The definition of two control groups were showed as follow: T2D patients whose accumulative diabetes prescription primarily consistent of insulin or metformin (lasting from the baseline date to the last follow‐up date), meanwhile other diabetic prescription accumulated less than 6 months will be classified as insulin‐only or metformin‐only group. The insulin group (control group 1) consisted of 36 146 T2D patients predominantly using insulin subcutaneous injections, while the metformin group (control group 2) comprised 124 371 T2D patients predominantly prescribed metformin. Comparative analyses were conducted to assess the risks of various adverse outcomes associated with GLP‐1 RAs compared to these two first‐line diabetic treatments during the follow‐up period. The detailed including criteria of pharmaceutical types were reported in Table 1S.

### 2.2 Definitions of outcomes and covariates

The outcomes of interest were symptoms that have been reported or hypothetically related to the GLP‐1 RAs usage in previous literature, animal studies, or clinical case reports,^5^, ^12^ which included six aspects of disease systems: digestive, renal, thyroid, skin and it is accessory organs, lymphatic, and hematopoietic. The *International Classification of Diseases Tenth Reversion* (ICD‐10) was used to identify cases in both outpatient and hospitalization records. Supplementally, a combination of ICD‐10 diagnosis, discharge diagnosis, imaging diagnosis, and regular expression for terminology recognition was applied to identify the outcomes. Detailed information for the identification of the outcomes was recorded in Table 2S.

All of the outcome events were limited to the newly onset incidents, defined as those diagnosed as diabetes only at baseline, whereafter detected as any of the aforementioned events of possible GLP‐1 RAs adverse effects. The recruited population, which was found within more than two interested outcome events, will be simultaneously recorded as correspondingly independent events. All incidence duration/survival times for outcomes of interest were operationalized as the number of months from the initial outpatient date at baseline to the occurrence of the first recorded hospitalization/outpatient events, whichever came first. The mean follow‐up time was 23.7 ± 18.5, 23.2 ± 18.3, and 24.8 ± 18.6 months for the GLP‐1 RAs, insulin‐only, and metformin‐only groups, respectively.

Covariates included demographic characteristics such as age (≤30, 30–49, 50–69, ≥70 years), sex (men, women, unspecified), education (high school and below, undergraduate and above, unspecified), health insurance (self‐pay, citizen health insurance, other), marital status (married, unmarried, divorced or widowed, unspecified), hypertension status (normal: <80/120 mmHg, prehypertension: 80–89/120–139 mmHg, hypertension: ≥90/140 mmHg), and BMI categories (18.5–24.9, 25.0–29.9, 30.0–39.9 kg/m^2^, unspecified) were extracted from a wide range of outpatient biochemical tests records, physical examination record, and chronic disease management records, whichever came first at baseline for propensity score matching (PSM) pairing and confounding adjustment.

### 2.3 Statistical analysis

Competing risks represent a critical aspect in the analysis of time‐to‐event data. In this study, the occurrence of acute side effects from GLP‐1 RAs may result in the discontinuation or interruption of both short‐term and long‐term usage, thus serving as competing risks. Therefore, the potential competing events were systematically defined as a binomial variable, activated by any incidence of specific acute symptoms, including diarrhea/emesis, dermatitis tetter, acne, dizziness, arrhythmia, etc. As such, to rigorously quantify the competing effects, a competing‐risks survival model was employed, allowing for the estimation of multivariable‐adjusted sub‐distribution hazard ratios (sHRs) and corresponding 95% confidence intervals (CIs).^13^ This approach ensures the minimization of the impact of competing events on the accurate estimation of potential adverse effects linked to GLP‐1 RAs, enhancing the integrity and validity of the study's findings.

Given the potential time‐effects caused by the duration of GLP‐1 RAs usage in T2D patients, the GLP‐1 RAs group was further divided into two groups based on 12 months of prescriptive usage. The duration of prescriptive usage was determined by the cumulative prescription coverage for each T2D patient. Two phases of risk assessments were performed to cross‐validate the solidity of our results. The phase 1 risk assessment was focused on the initial evaluation among the natural T2D population without biases‐pairing. To eliminate the potential biases, phase 2 risk assessment was conducted using a 1:2 paired population by PSM method on variables including age, sex, education, marital status, and BMI. In the additional analyses, we further conducted subgroup risk assessment among four different GLP‐1 RA agents, including liraglutide, loxenatide, lixisenatide, and semaglutide which subgrouped by the predominant GLP‐1 RA agent (same agent lasting from the baseline date to the last follow‐up, with any accumulative prescription change of other GLP‐1 RA agent less than 6 months) use during our research period. Due to limited sample sizes, no further subgrouping by usage duration was done for loxenatide, lixisenatide, and semaglutide.

This study was approved by the Ethics Committees of the Southern University of Science and Technology (Ethical Approval ID: 20210067). All the statistical analyses were performed by SAS 9.4 software, version 9.4 (SAS Institute Inc. NC, U.S.) with two‐sided tests under a significance level of *ρ* < 0.05.

## 3 RESULTS

Table 3S presented the baseline demographic characteristics of the study population in risk assessment phase 1. Significant differences were observed regarding age, educational level, marital status, BMI, and fasting glucose level. A notably higher proportion of obesity (BMI 25.0–29.9: 30.51%, BMI 30.0–39.9:13.21%) was observed within the GLP‐1 RAs group, compared with other treatment groups. Also, the GLP‐RAs group demonstrated a substantially higher proportion of higher education levels and better support from health insurance. Table 1 presented the demographic characteristics following PSM among the GLP‐1 RAs user group, insulin injection group, and metformin group. The results displayed an evenly distributed set of demographic covariates across groups, suggesting a successful pairing.

**TABLE 1:** Demographic data after propensity score matching (PSM) among glucagon‐like peptide‐1 receptor agonists (GLP‐1 RAs), insulin, and metformin groups.

Demographic	1:2 paired population, *n* (%)		1:2 paired population, *n* (%)	
	GLP‐1 RA	Insulin (%)	GLP‐1 RA	Metformin (%)
	*N1* = 7383	*N2* = 13 827	*N1* = 7680	*N1* = 15 163
Sex, *n* (%)				
Men	4190 (56.75)	7859 (56.84)	4362 (56.80)	8618 (56.84)
Women	3188 (43.18)	5960 (43.10)	3312 (43.13)	6536 (43.10)
Unspecific	5 (0.07)	8 (0.06)	6 (0.07)	9 (0.06)
Age (years), *n* (%)				
<30	302 (4.09)	499 (3.61)	353 (4.60)	564 (3.72)
30~	2600 (35.22)	4538 (32.82)	2816 (36.67)	5586 (36.84)
50~	2529 (34.25)	4899 (35.43)	2555 (33.27)	5104 (33.66)
≥70	436 (5.91)	859 (6.21)	440 (5.73)	877 (5.78)
Unspecified	1516 (20.53)	3032 (21.93)	1516 (19.74)	3032 (20.00)
Education, *n* (%)				
High school and below	3141 (42.54)	5923 (42.84)	3217 (41.89)	6393 (42.16)
Undergraduate and above	1760 (23.84)	2941 (21.27)	1981 (25.80)	3837 (25.31)
Unspecified	2482 (33.62)	4963 (35.89)	2481 (32.31)	4933 (32.53)
Health insurance, *n* (%)				
Self‐pay	1784 (24.16)	3454 (24.98)	1807 (23.53)	3576 (23.58)
Citizen health insurance	5542 (75.06)	10 273 (74.30)	5809 (75.64)	11 466 (75.62)
Other	57 (0.77)	100 (0.72)	64 (0.83)	121 (0.80)
Marriage, *n* (%)				
Unmarried	185 (2.51)	277 (2.00)	255 (3.32)	398 (2.62)
Married	4657 (63.08)	8481 (61.34)	4884 (63.59)	9717 (64.08)
Divorced or widowed	59 (0.80)	106 (0.77)	60 (0.78)	115 (0.76)
Unspecified	2482 (33.62)	4963 (35.89)	2481 (32.30)	4933 (32.53)
BMI, *n* (kg/m^2^, %)				
18.5–24.9	1850 (25.06)	3686 (26.66)	1854 (24.14)	3696 (24.38)
25.0–29.9	2327 (31.52)	4227 (30.57)	2352 (30.63)	4655 (30.70)
30.0–39.9	710 (9.62)	925 (6.69)	979 (12.75)	1852 (12.21)
Unspecified	2496 (33.81)	4989 (36.08)	2495 (32.49)	4960 (32.71)
Fasting plasma glucose (mmol/L)^a^	7.29 ± 2.53	7.49 ± 2.65	7.27 ± 2.50	7.57 ± 2.54
Hemoglobin A1c (%)^a^	6.79 ± 5.44	6.55 ± 4.67	6.82 ± 5.37	6.41 ± 4.67

^a^ The fasting plasma glucose (mmol/L) and hemoglobin A1c (percentage %) were presented as the mean ± standard deviation among different groups. The missing rates for the Fasting plasma glucose (FPG) and Hemoglobin A1c (HbA1c) were 33.22% and 91.27%, respectively.

### 3.1 GLP‐1 RAs versus insulin

In risk assessment phase 1 without biases‐pairing (Figure 1S), substantially increased incidence risks of pancreatitis, gastric ulcer, acute nephritis, and leukemia were observed in both short‐term and/or long‐term usage of GLP‐1 RAs when compared with insulin subcutaneous injection group.

**FIGURE 2:**
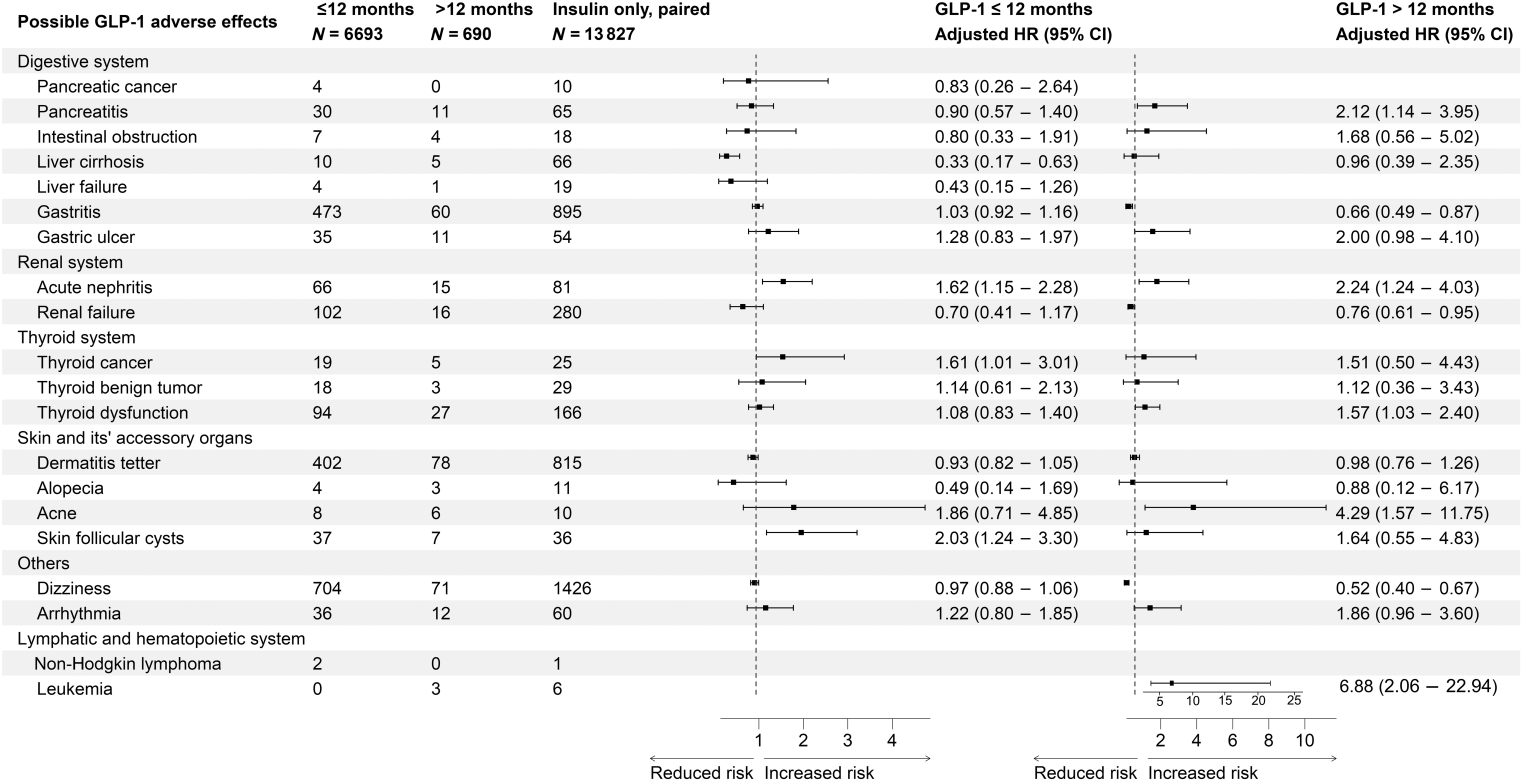
Incidence risks of potential glucagon‐like peptide‐1 receptor agonists (GLP‐1 RAs) adverse effects in comparison to insulin subcutaneous injections after propensity score matching (PSM), GLP‐RAs group stratified by 12 months usage. The final results of competing risks model were presented as sub‐distribution hazard ratios (sHRs) with 95% confidence intervals (CIs) for each corresponding GLP‐1 RAs group which was stratified by the duration of usage.

After PSM (Figure 2), The increased incidence risks of acute nephritis (sHR: 1.62, 95% CI: 1.15–2.28), thyroid cancer (sHR: 1.61, 95% CI: 1.01–3.01), and skin follicular cysts (sHR: 2.03, 95% CI: 1.24–3.30) were observed when using GLP‐1 RAs under 12 months; For over 12 months of GLP‐1 RAs usage group, significantly increased incidence risks of acute nephritis (sHR: 2.24, 95% CI: 1.24–4.03), thyroid dysfunction (sHR: 1.57, 95% CI: 1.03–2.40), and acne (sHR: 4.29, 95% CI: 1.57–11.75) were also observed. Notably, pancreatitis (sHR: 2.12, 95% CI: 1.14–3.95) and leukemia (sHR: 6.88, 95% CI: 2.06–22.94) were exclusively associated with long‐term GLP‐RAs (>12 months) usage when compared with the insulin group.

### 3.2 GLP‐1 RAs versus metformin

When compared with the unpaired metformin group (Figure 2S), increased incidence risks of pancreatitis, gastric ulcer, acute nephritis, renal failure, thyroid cancer/dysfunction, and acne were evident in both short‐term and/or long‐term usage of GLP‐1 RAs. However, significant elevated incidence risks for intestinal obstruction, liver cirrhosis, and leukemia were only observed after long‐term usage of GLP‐1 RAs.

**FIGURE 3:**
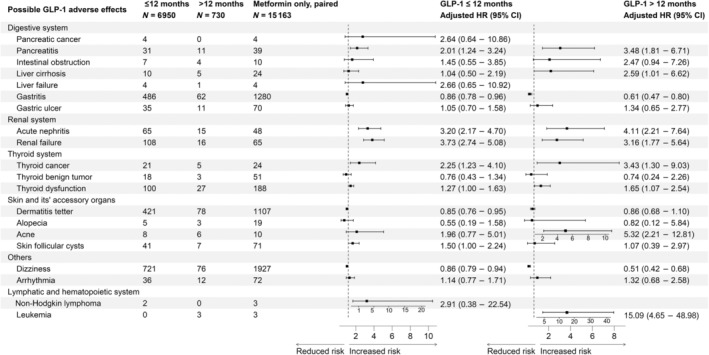
Incidence risks of potential glucagon‐like peptide‐1 receptor agonists (GLP‐1 RAs) adverse effects in comparison to metformin use after propensity score matching (PSM), GLP‐RAs group stratified by 12 months. The final results of competing risks model were presented as sub‐distribution hazard ratios (sHRs) with 95% confidence intervals (CIs) for each corresponding GLP‐1 RAs group which was stratified by the duration of usage.

After PSM (Figure 3), GLP‐1 RAs use under 12 months group appeared to be associated with increased incidence risks of pancreatitis (sHR: 2.01, 95% CI: 1.24–3.24), acute nephritis (sHR: 3.20, 95% CI: 2.17–4.70), renal failure (sHR: 3.73, 95% CI: 2.74–5.08), thyroid cancer (sHR: 2.25, 95% CI: 1.23–4.10), thyroid dysfunction (sHR: 1.27, 95% CI: 1.00–1.63), and skin follicular cysts (sHR: 1.50, 95% CI: 1.00–2.24); While after 12 months of GLP‐1 RAs usage, the incidence risks of pancreatitis (sHR: 3.48, 95% CI: 1.81–6.71), acute nephritis (sHR: 4.11, 95% CI: 2.21–7.64), renal failure (sHR: 3.16, 95% CI: 1.77–5.64), thyroid cancer (sHR: 3.43, 95% CI: 1.30–9.03), thyroid dysfunction (sHR: 1.65, 95% CI: 1.07–2.54), and acne (sHR: 5.32, 95% CI: 2.21–12.81) were still evident and appeared to be higher than the short‐term usage. Moreover, significantly increased incidence risks of leukemia (sHR: 15.09, 95% CI: 4.65–48.98) were only found after long‐term (≥12 months) GLP‐1 RAs usage.

### 3.3 Different GLP‐1 RA agents and adverse health outcomes

Subgroup analyses were performed on four GLP‐1 RA agents: liraglutide, loxenatide, lixisenatide, and semaglutide. Detailed demographic data for each 1:2 PSM can be found in Tables 4S–7S.

For liraglutide (Figures 3S and 4S), substantially increased incidence risks of pancreatitis, acute nephritis, thyroid cancer, thyroid dysfunction, and leukemia were observed in comparison to both insulin and metformin groups. While the elevated incidence risk of intestinal obstruction (>12 months) and renal failure were only observed when compared with the metformin group; In the loxenatide group (Figures 5S and 6S), only gastritis was found to be statistically significant when compared to both the insulin (sHR: 2.31, 95% CI: 1.36–3.93) and metformin groups (sHR: 1.63, 95% CI: 1.00–2.66); Moreover, the risks of pancreatitis and acute nephritis approached borderline significance when compared to insulin group and metformin group for the lixisenatide group (Figures 7S and 8S), Additionally for the semaglutide group (Figures 9S and 10S), only the renal failure demonstrated significantly increased incidence risks when compared to the metformin group.

## 4 DISCUSSION

GLP‐1 RAs have received significant attention worldwide due to their favorable effects on glycemic and body weight control. Our findings, however, provided a comprehensive view of the health risks, both in terms of short‐term and long‐term in different organ systems, associated with GLP‐1 RAs therapy in comparison to first‐line diabetes medications.

Previous studies have reported that the initial gastrointestinal symptoms such as nausea, diarrhea, vomiting, and dyspepsia were more pronounced at the onset of GLP‐1 RAs treatment,^14^ but generally diminished.^15^, ^16^ However, we observed notably increased incidence risks of gastric ulcer and intestinal obstruction (Supporting Information, Figure 1S and 2S) associated with GLP‐1 RAs use without PSM, particularly after 1 year of treatment. These results aligned with the recent research by Faillie et al.^17^ and the case report from Mathew et al.^18^ which demonstrated a significantly elevated incidence risk of intestinal obstruction in patients using GLP‐1 RAs compared with those on SGLT‐2 inhibitors. Notably, they also highlighted the time‐dependent effects of GLP‐1 RAs, with the highest HR of 3.48 that was observed after 1.6 years of use. Another U.S. population‐based study also observed a significantly increased incidence risk of bowel obstruction (HR: 4.22) associated with GLP‐RAs, as compared with bupropion‐naltrexone users for weight loss.^19^ Although the underlying mechanisms for GLP‐1 RA‐induced suppression of intestinal motility remain incompletely understood, plausible pathways may involve vagal cholinergic routes or direct interactions with central and enteric GLP‐1 receptors. At the presynaptic level, prolonged GLP‐1 RA use may modulate nitric oxide release, inhibiting neurotransmission within the enteric nervous system.^20^, ^21^ These mechanisms could be further linked to appetite regulation, satiety, reduced gastric acid secretion, and delayed gastric and intestinal motility, thereby contributing to an elevated risk of gastrointestinal diseases.

Our study also found an increased incidence risk of pancreatitis in the GLP‐1 RAs group when compared to both insulin and metformin groups. In fact, concerns have also been expressed regarding the potential association of GLP‐1 RAs treatment with pancreatic inflammation and pancreatitis.^22^, ^23^, ^24^ Clinical studies have indicated deleterious effects of GLP‐1 RAs on pancreatic tissue.^25^, ^26^ For instance, a recent U.S. population‐based study reported a significantly increased incidence risks of pancreatitis (HR: 9.09) associated with GLP‐1 RAs treatment for weight loss.^19^ Also, another study including 90 T2D patients on GLP‐1 RAs or dipeptidyl peptidase 4 inhibitors (DPP4i) reported that 36% of GLP‐RAs users had elevated serum amylase or lipase levels,^27^ and the large‐scale Liraglutide Effect and Action in Diabetes: Evaluation of Cardiovascular Outcome Results (LEADER) trial reported that after usage of GLP‐1 RAs, over 22.7% of patients had elevated enzyme levels at baseline^28^; although a meta‐analysis of trials indicated a slight, albeit nonsignificant elevation in pancreatitis risk associated with GLP‐1 RAs.^29^ However, some studies did not corroborate this elevated risk.^30^ This inconclusive evidence has prompted the safety investigation of these drugs.^31^ As T2D and hypertriglyceridemia independently predispose patients to pancreatitis, these potential confounding factors warrant consideration in evaluating any association between GLP‐1 RAs and pancreatitis. Given these complexities and until more definitive evidence is available, caution and strict monitoring should be prudent when prescribing GLP‐1 RAs to patients with risk factors for pancreatitis.

For renal systems, elevated incidence risks of acute nephritis and renal failure were both observed when compared with the metformin group, or only increased incidence risks of acute nephritis compared with the insulin injection group. This observation may be substantially influenced by the stage of T2D, given that patients in the advanced stages of T2D are more commonly prescribed insulin injections. Simultaneously, the renal impairment is more prevalent within this patient group.^32^, ^33^ Our results may highlight the importance of strict monitoring of kidney function for GLP‐1 RAs users at the treatment's commencement, particularly for the early‐moderate stage T2D patients. Hypothetically, the GLP‐1 RAs may not be suitable for all T2D patients, because the GLP‐1 receptor agonist may be linked to acute kidney injury, predominantly through prerenal acute failure caused by factors, such as drug‐induced nausea, vomiting, and reduced fluid intake.^34^, ^35^ Also, other risk factors like cardiac failure, hypertension, or the use of nephrotoxic drugs associated with diabetes may worsen kidney function.^5^, ^34^, ^36^ Thus, physicians should still consider the potential risks of acute nephritis and renal failure in administering GLP‐1 RAs, particularly for individuals with uncontrolled T2D, polyuria, polydipsia, or those prone to volume depletion.^35^, ^37^, ^38^, ^39^

We also observed significantly increased incidence risks of thyroid cancer and thyroid dysfunction in the GLP‐1 RAs patients, and these risks also increased in a manner directly proportional to the duration of GLP‐1 RAs therapy. Indeed, concerns have emerged regarding a potential adverse association between GLP‐1 RAs and medullary thyroid cancer, largely based on rodent studies, where liraglutide and exenatide were linked to the development of thyroid C‐cell tumors at supratherapeutic doses.^40^, ^41^ A systematic review involving 45 clinical trials also showed GLP‐1 RAs were associated with a 28% increased risk of overall thyroid disorders.^42^ Another nested case–control study based on the French national health care insurance system (SNDS) database also demonstrated a substantially increased incidence risk of overall thyroid cancer (HR: 1.58, 95% CI: 1.27–1.95),^43^ compared with T2D patients without GLP‐1 RAs users. Moreover, real‐world studies from the Food and Drug Administration Adverse Event Reporting System also reported significantly increased reports ratios of thyroid cancer (proportional reporting ratio, PRR: 27.43) and papillary thyroid cancer (PRR: 8.68)^44^, ^45^ associated with GLP‐1 RAs and DDP4i use. Given that neoplastic and hyperplastic lesions of thyroid C cells express the GLP‐1 receptor, the consequence of long‐term increased GLP‐1 signaling on these GLP‐1 receptor‐expressing cells in the human thyroid gland required critical attention.^46^

Prior research has highlighted skin‐related adverse reactions associated with GLP‐1 RAs injection, chiefly characterized by rashes, erythema, or itching at the injection site,^47^ which is largely similar to our findings. GLP‐1 RAs' adverse cutaneous effects primarily pertain to the injection site, with more prevalent reactions noted with long‐acting GLP‐1 RAs,^12^ which are theoretically attributed to the drug's polymer microsphere formulation.^47^, ^48^ There have also been sporadic case reports associating exenatide use with the development of panniculitis^49^, ^50^; however, the underlying mechanism was largely unknown. Clinical practitioners may need to be attentive to adverse skin reactions when prescribing GLP‐1 RAs.

Of note was that we also observed an increased incidence risk of leukemia in the GLP‐1 RAs group after 12 months of usage. This finding should be interpreted cautiously due to the random bias caused by a relatively low incidence rate and cases of leukemia in our study. To the best of our knowledge, no related research has explored the link between GLP‐1 RAs usage and leukemia. Consequently, a compelling need in the future is strongly warranted to validate our findings.

## 5 LIMITATIONS

While our study offers valuable insights into the potential adverse effects of GLP‐1 RAs in a real‐world scenario, we acknowledge several limitations inherent in our methodology. First, the reliance on data exclusively from Shenzhen city in China may introduce potential selection bias, further studies in generalizing our findings should be conducted among more diverse populations. Second, the presence of missing data, particularly in biochemical parameters, posed a challenge to the comprehensive assessment of GLP‐1 RAs adverse effects. Also, despite our meticulous efforts to control for confounding factors, the missing data on certain lifestyle variables might result in residual confounding. Third, GLP‐1 RAs users at advanced disease stages may contribute to adverse events, potentially stemming from the T2D severity level itself rather than pharmacological effects. However, our study mitigated this potential bias by excluding patients with symptoms of advanced diabetes, such as chronic kidney disease, CVDs, and cancer, which may further reduce this bias.

Additionally, by employing two comparison groups, categorized by metformin and insulin monotherapy, allowed for comparisons across different disease severities. To some extent, the former group represented the mild stage of T2D, whereas the latter group consisted of the severe state of T2D. This approach may offer insights into early and moderate disease durations, thus partially addressing the bias. Meanwhile, our case and control groups were categorized by predominant medication use may allow us to minimize the interference of different medications used simultaneously. Nevertheless, this approach may differ from real‐world settings where monotherapy for T2D is less common. Thus, future studies are strongly warranted to better gauge the role of disease severity in the association between GLP‐1 RAs and potential adverse effects.

## 6 CONCLUSION

GLP‐1 RAs use in T2D patients may be associated with a wide range of potential adverse health outcomes among the Chinese population in a real‐world scenario. The magnitude and types of risks appear to differ based on the duration of GLP‐1 RAs usage. Cautions regarding the risk–benefit balance should be considered in clinical practice.

## AUTHOR CONTRIBUTIONS

Zhiyuan Cheng, Shuang Wang, Zheng Jing, Fu‐rong Li, and Dongfeng Gu made substantial contributions to the study conception and design, acquisition of data, or analysis and interpretation of the data. Fengchao Liang and Dongfeng Gu obtained grant funding. All authors drafted (Zhiyuan Cheng) or revised (Furong Li, Cheng Jin, Chunbao Mo, Xia Li, Fengchao Liang, Jinkui Yang, and Dongfeng Gu) the article for important intellectual content. All co‐authors approved the final version to be published.

## FUNDING INFORMATION

This work was supported by the R&D project of Pazhou Lab (Huangpu) under Grant 2023K0610, the National Natural Science Foundation of China (12126602, 82030102, 42107465), Shenzhen Medical Academy of Research and Translation (C2302001), Shenzhen Science and Technology Innovation Committee (ZDSYS20200810171403013), and the Ministry of Science and Technology of China (2022YFC3702703).

## CONFLICT OF INTEREST STATEMENT

The authors declare no conflicts of interest.

## Supporting information


**Data S1.** Supporting Information.

## Data Availability

The datasets generated during the current study were not publicly available due to the privacy protection of all enrolled participants.
