# Supplementary material for: The potential adverse effects of hypodermic glucagon‐like peptide ‐1 receptor agonist on patients with type 2 diabetes: A population‐based study
Source: J Diabetes. 2024 Oct 22;16(10):e70013. doi: 10.1111/1753-0407.70013 (PMC11494487; doi:10.1111/1753-0407.70013)
Supplement: Supplementary file 1 — Data S1. Supporting Information. [file JDB-16-e70013-s001.docx]

Appendices information for

**The potential adverse effects of hypodermic GLP-1 receptor agonist on patients with type 2 diabetes: a population-based study**

Number of pages: 18

Number of tables: 7

Number of figures:10

Number of texts: 11

**Contents:**

Supplement Table 1S Coverage of selected pharmaceutical types Page 2

Supplement Table 2S Including criteria of potential adverse effects Page 3

Supplement Table 3S Demographic data of risk assessment phase one Page 4

Supplement Figure 1S Results of phase 1 risks assessment: vs. insulin Page 5

Supplement Figure 2S Results of phase 1 risks assessment: vs. metformin Page 6

Supplement Table 4S Demographic of Liraglutide and paired control groups Page 7

Supplement Figure 3S-4S Risk asseseement of Liraglutide Page 8-9

Supplement Table 5S Demographic of Loxenatide and paired control groups Page 10

Supplement Figure 5S-6S Risk asseseement of Loxenatide Page 11-12

Supplement Table 6S Demographic of Lixisenatide and paired control groups Page 13

Supplement Figure 7S-8S Risk asseseement of Lixisenatide Page 14-15

Supplement Table 7S Demographic of Semaglutide and paired control groups Page 16

Supplement Figure 9S-10S Risk asseseement of Semaglutide Page 17-18

| **Table 1S. Coverage of pharmaceutical types/trade names in cases and control groups** | | | | |
| --- | --- | --- | --- | --- |
| **GLP-1 receptor agonist** |  | **Metformin** |  | **Insulin (300 u)** |
| Liraglutide (Intermediate) |  | Metformin |  | Intermediate-acting insulin |
| Lixisenatide (Intermediate) |  | Metformin (sustained release) |  |  |
| Loxenatide (Long-acting) |  | Metformin (enteric coatel) |  | Long-acting insulin |
| Semaglutide (Long-acting) |  | Glucophage |  |  |
|  |  | Phenformin |  |  |

**Table 2S. Included and filters of possible or reported adverse effects of GLP-1 RAs**

| **Categories of possible GLP-1 side-effects** | **ICD-10** | **Regular expression match** |
| --- | --- | --- |
| **Digestive system** |  |  |
| Pancreatic cancer | C25 | ‘/Pancreatic (cancer\|carcinoma)/’ |
| Pancreatitis | K85, K86 | ‘/(acute\|chronic)\s+pancreatitis/’ |
| Intestinal obstruction | K56 | ‘/Intestinal\s+obstruction/' |
| Liver cirrhosis | K74 | ‘/Liver cirrhosis/' |
| Liver failure | K72 | ‘/Liver failure/' |
| Gastritis | K29 | ‘/acute\/chronic\s+Gastritis/’ |
| Gastric ulcer | K25 | ‘/Gastric ulcer/' |
| **Renal system** |  |  |
| Acute nephritis | N00-N01,N03-N06, N08 | ‘/Acute nephritis\|Nephrotic syndrome/’ |
| Renal failure | N17-N19 | ‘/Chronic kidney disease\|Acute kidney failure/ ' |
| **Thyroid system** |  |  |
| Thyroid cancer | C73, C75 | ‘/Thyroid (papillary\|follicular\|medullary) (cancer\|carcinoma)/’ |
| Thyroid benign tumor | D34, E04 | ‘/Thyroid benign tumor\|Thyroid nodules/’ |
| Thyroid dysfunction | E03-E07 | ‘/Thyroid dysfunction  \|Hyperthyreosis\|Hypothyroidism/’ |
| **Skin and it’s accessory organs** |  |  |
| Dermatitis tetter | L20-L30,L50-L54 | ‘/Dermatitis\|Cutitis\|Tetter\|Erythra\|Urticaria/’ |
| Alopecia | L62-L66 | ‘/Alopecia/' |
| Acne | L70,L71 | ‘/Acne/' |
| Skin follicular cysts | L72,L73 | ‘/Skin follicular cysts\|Follicular disorders/’ |
| **Lymphatic and hematopoietic system** |  |  |
| Non-Hodgkin lymphoma | C82,C83,C85 | ‘/Follicular lymphoma\|Non-follicular lymphoma\|T\/NK-cell lymphomas/’ |
| Leukemia | C91-C95 | ‘/Leukemia/' |
| **Others** |  |  |
| Dizziness | H81, R42 | ‘/Vestibular\|Dysequilibrium\|Dizziness\|Vertigo/' |
| Arrhythmia | I47,I49 | ‘/Tachycardia\|Arrhythmia\|Dysrhythmia' |

**Table 3S Demographic characteristics of the phase one risk assessment at baseline.**

| **Demographic** | **Baseline data, n (%)** | | |  |
| --- | --- | --- | --- | --- |
|  | **GLP-1 agonists only** | **Insulin injection only (%)** | **Metformin**  **only (%)** | |
|  | **N=7,746** | **N=36,146** | **N=124,371** | |
| **Sex, n (%)** |  |  |  | |
| Men | 4,389 (56.66) | 19,869 (54.97) | 68,533 (55.10) | |
| Women | 3,344 (43.17) | 16,206 (44.83) | 55,680 (44.77) | |
| Unspecific | 13 (0.17) | 71 (0.20) | 158 (0.13) | |
| **Age (years), n (%)** |  |  |  | |
| <30 | 391 (5.05) | 1,458 (4.03) | 1,008 (0.81) | |
| 30~ | 2,834 (36.59) | 9,699 (26.83) | 30,926 (24.87) | |
| 50~ | 2,561 (33.06) | 14,933 (41.31) | 58,669 (47.17) | |
| ≥70 | 443 (5.72) | 3,737 (10.34) | 11,103 (8.93) | |
| Unspecified | 1,517 (19.58) | 6,319 (17.48) | 22,655 (18.22) | |
| **Education, n (%)** |  |  |  | |
| High school and below | 3,237 (41.79) | 18,775 (51.94) | 80,010 (64.33) | |
| Undergraduate and above | 2019 (26.07) | 5,656 (15.65) | 14,852 (11.94) | |
| Unspecified | 2490 (32.15) | 11,715 (32.41) | 29,509 (23.73) | |
| **Health insurance, n (%)** |  |  |  | |
| Self-pay | 1,819 (23.51) | 14,256 (39.47) | 55,041 (44.28) | |
| Citizen health insurance | 5,850 (75.60) | 21,525 (59.60) | 68,326 (54.96) | |
| Other | 69 (0.89) | 334 (0.92) | 948 (0.76) | |
| **Marriage, n (%)** |  |  |  | |
| Unmarried | 285 (3.68) | 927 (2.56) | 1,191 (0.96) | |
| Married | 4,905 (63.32) | 23,012 (63.66) | 91,997 (73.97) | |
| Divorced or widowed | 66 (0.85) | 488 (1.35) | 1,659 (1.33) | |
| Unspecified | 2,490 (32.15) | 11,719 (32.42) | 29,524 (23.74) | |
| **BMI, n (kg/m^2^,%)** |  |  |  | |
| 18.5~24.9 | 1,857 (23.97) | 15,762 (43.61) | 51,772 (41.63) | |
| 25.0~29.9 | 2,363 (30.51) | 7,436 (20.57) | 36,688 (29.50) | |
| 30.0~39.9 | 1,023 (13.21) | 1,100 (3.04) | 6,053 (4.87) | |
| Unspecified | 2,503 (32.31) | 11,848 (32.78) | 29,858 (24.01) | |
| **Fasting plasma glucose (mmol/L)*** | 7.27±2.51 | 7.59±2.79 | 7.68±2.63 | |
| **Hemoglobin A1c (%)*** | 6.82±6.35 | 6.33±4.27 | 6.69±5.30 | |

Note: The fasting plasma glucose (mmol/L) and hemoglobin A1c (percentage %) were presented as the mean ± standard deviation among different groups. The missing rates of the FPG were 32.95%, 32.66%, and 24.13% among GLP-1 RAs, insulin only, and metformin groups, respectively; The missing rates of the HbA1c were 90.52%, 91.79%, and 90.45% among GLP-1 RAs, insulin only and metformin only groups, respectively.

Table 3S presented the baseline demographic characteristics of the study population in risk assessment phase one. A comparison between the GLP-1 RAs and 2 control groups revealed significant differences in the distribution of age, educational level, marital status, BMI, and fasting glucose level.


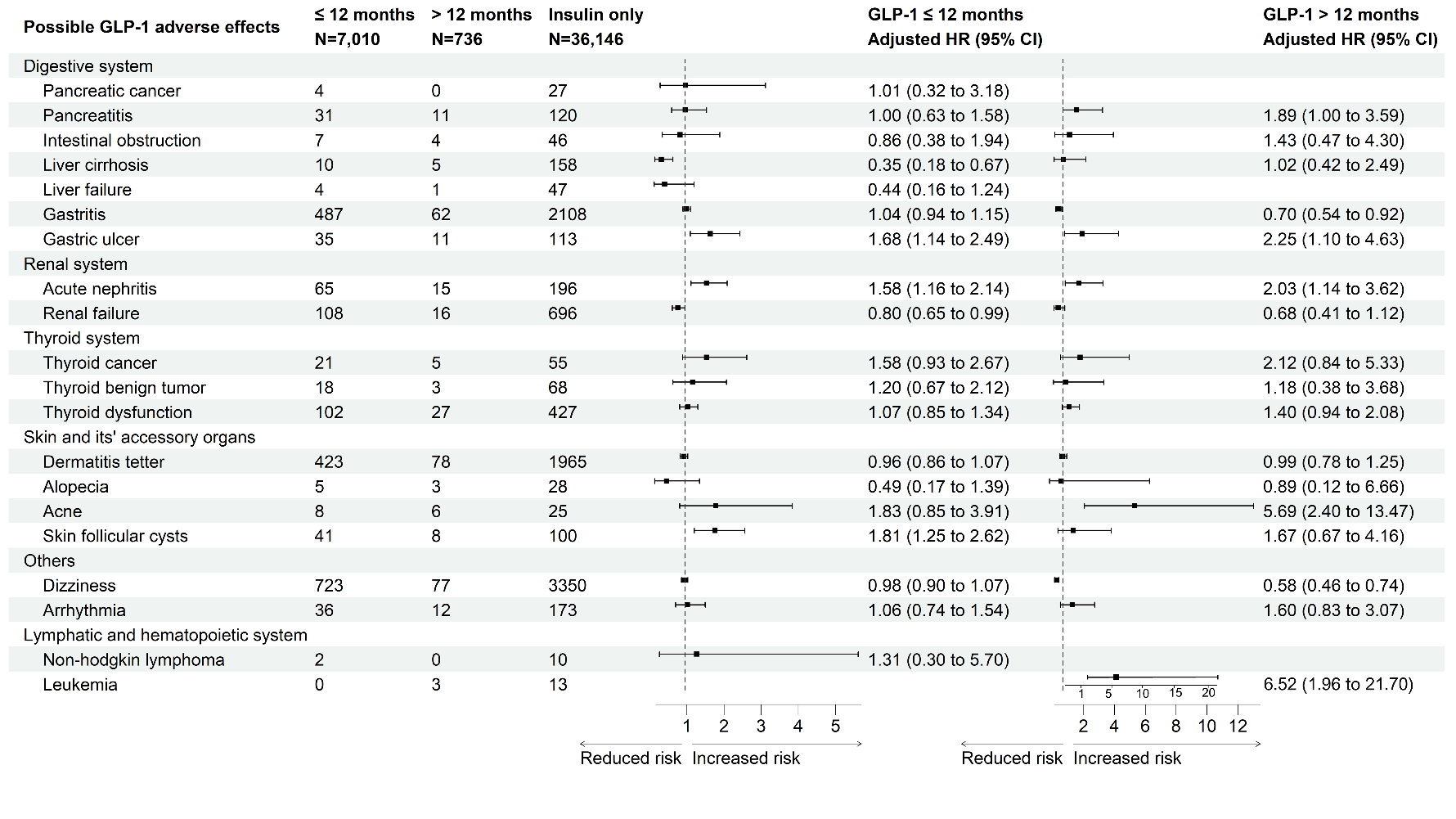
**Figure 1S Incidence risks of potential GLP-1 RAs adverse effects in comparison to the insulin subcutaneous injections (unpaired), GLP-RAs group stratified by 12 months usage**

Figure 1S presented the adjusted sub-distributional HR of potential adverse outcomes of GLP-1 RAs usage without bias-pairing but fully adjusted by potential confounding factors (age, sex, education, health insurance, marital status, hypertension status, and BMI categories). When compared to the T2D patients only with insulin treatment, gastric ulcer, acute nephritis, acne, and skin follicular cysts were observed with significantly increased incidence risks in both under and over 12-months usage populations. Notably, the increased risk of pancreatitis (sHR:1.89, 95% CI:1.00-3.59) and leukemia was only observed in the GLP-1 RAs ≥12 months group (sHR:6.52, 95% CI:1.96-21.70).


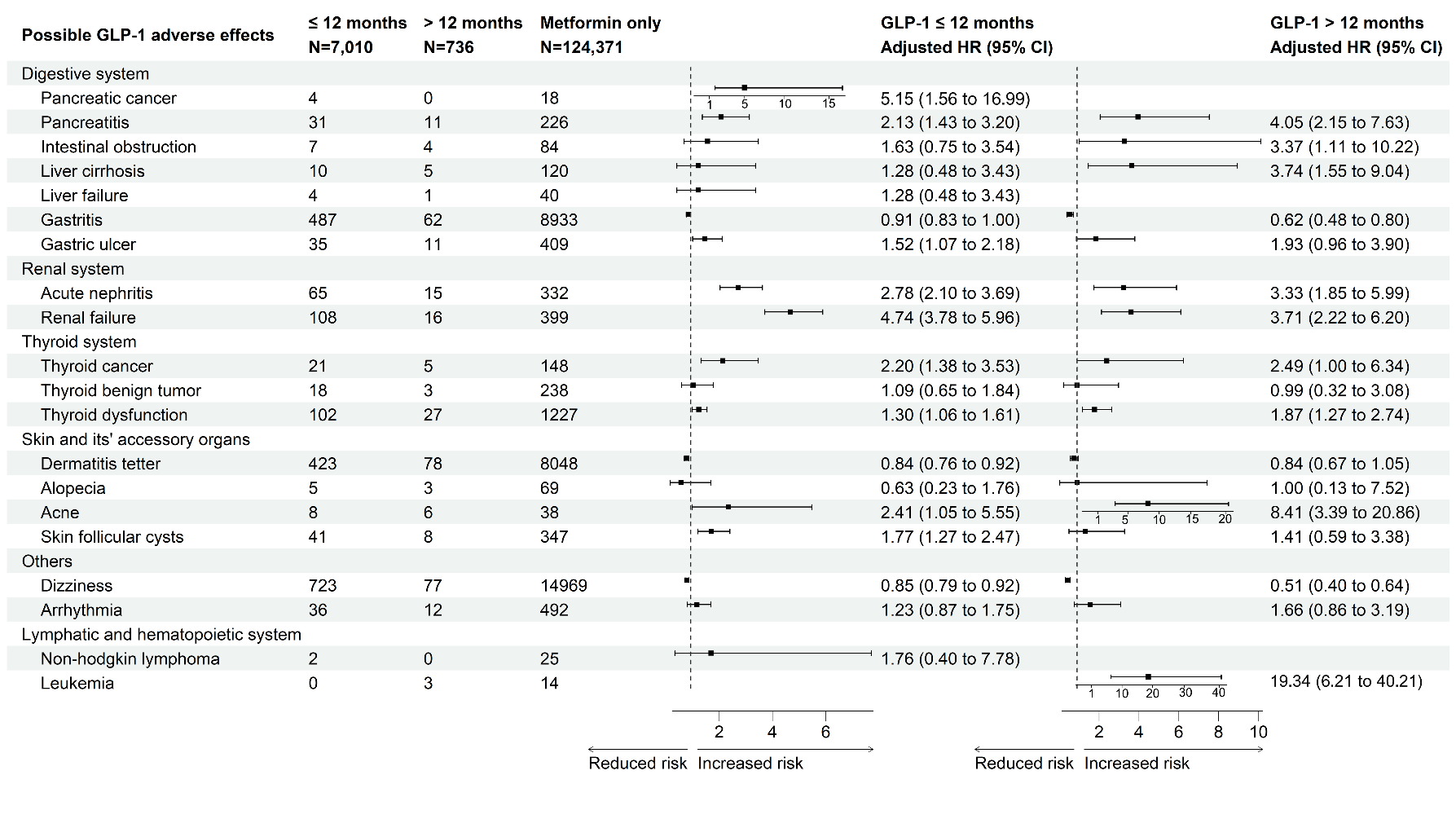
**Figure 2S.** **Incidence risks of potential GLP-1 RAs adverse effects in comparison to the metformin (unpaired), GLP-RAs group stratified by 12 months usage**

Figure 2S presented the potential adverse effects of GLP-1 RAs usage compared with the metformin-only group without bias-pairing but fully adjusted by potential confounding factors (age, sex, education, health insurance, marital status, hypertension status, and BMI categories). Various adverse outcomes demonstrated significant association with GLP-1 RAs use, including pancreatic cancer, pancreatitis, gastric ulcer, acute nephritis, renal failure, thyroid cancer, thyroid dysfunction, etc. Meanwhile, increased incidence risks of intestinal obstruction (sHR:3.37, 95% CI:1.11-10.22), liver cirrhosis (sHR:3.74, 95% CI:1.55-9.04), and leukemia (sHR: 19.34, 95% CI: 6.21-40.21) were exclusively observed when GLP-1 RAs usage over 12 months.

**Table 4S. Demographic data after PSM among Liraglutide, Insulin, and Metformin groups**

| **Demographic** | **1:2 paired population, n (%)** | |  | **1:2 paired population, n (%)** | |
| --- | --- | --- | --- | --- | --- |
|  | **Liraglutide** | **Insulin (%)** |  | **Liraglutide** | **Metformin (%)** |
|  | **N1=6,135** | **N2=11,699** |  | **N1=6,374** | **N1=12,556** |
| **Sex, n (%)** |  |  |  |  |  |
| Men | 3433 (55.96) | 6599 (56.41) |  | 3576 (56.10) | 5454 (43.44) |
| Women | 2697 (43.96) | 5092 (43.53) |  | 2793 (43.82) | 7095 (56.51) |
| Unspecific | 5 (0.08) | 8 (0.07) |  | 5 (0.08) | 7 (0.06) |
| **Age (years), n (%)** |  |  |  |  |  |
| <30 | 252 (4.11) | 416 (3.56) |  | 292 (4.58) | 504 (4.01) |
| 30~ | 2017 (32.88) | 3729 (31.87) |  | 2190 (34.36) | 4348 (34.63) |
| 50~ | 2087 (34.02) | 4080 (34.87) |  | 2110 (33.10) | 4215 (33.57) |
| ≥70 | 404 (6.59) | 795 (6.80) |  | 407 (6.39) | 808 (6.44) |
| Unspecified | 1375 (22.41) | 2679 (22.90) |  | 1375 (21.57) | 2681 (21.35) |
| **Education, n (%)** |  |  |  |  |  |
| High school and below | 2616 (42.64) | 5017 (42.88) |  | 2673 (41.94) | 5318 (42.35) |
| Undergraduate and above | 1439 (23.46) | 2596 (22.19) |  | 1622 (25.45) | 3151 (25.10) |
| Unspecified | 2080 (33.90) | 4086 (32.93) |  | 2079 (32.62) | 4087 (32.55) |
| **Health insurance, n (%)** |  |  |  |  |  |
| Self-pay | 1522 (24.81) | 2907 (24.85) |  | 1541 (24.18) | 2986 (23.78) |
| Citizen health insurance | 4570 (74.49) | 8714 (74.48) |  | 4784 (75.05) | 9480 (75.50) |
| Other | 43 (0.70) | 78 (0.67) |  | 49 (0.77) | 90 (0.72) |
| **Marriage, n (%)** |  |  |  |  |  |
| Unmarried | 152 (2.48) | 229 (1.96) |  | 211 (3.31) | 339 (2.70) |
| Married | 7302 (62.42) | 3860 (62.92) |  | 4041 (63.40) | 8048 (64.10) |
| Divorced or widowed | 43 (0.70) | 82 (0.70) |  | 43 (0.67) | 82 (0.65) |
| Unspecified | 2080 (33.90) | 4086 (32.93) |  | 2079 (32.62) | 4087 (32.55) |
| **BMI, n (kg/m^2^, %)** |  |  |  |  |  |
| 18.5~24.9 | 1512 (24.65) | 3018 (25.80) |  | 1515 (23.77) | 3019 (24.04) |
| 25.0~29.9 | 1941 (31.64) | 3694 (31.58) |  | 1961 (30.77) | 3885 (20.94) |
| 30.0~39.9 | 589 (9.6) | 876 (7.49) |  | 806 (12.65) | 1540 (12.27) |
| Unspecified | 2093 (34.12) | 4111 (35.114) |  | 2092 (32.82) | 4112 (32.75) |
| **Fasting plasma glucose (mmol/L) *** | 7.26±2.53 | 7.49±2.58 |  | 7.24±2.50 | 7.53±2.50 |
| **Hemoglobin A1c (%) *** | 6.91±5.84 | 6.46±4.17 |  | 6.93±6.78 | 6.35±4.90 |

Note: * The fasting plasma glucose (mmol/L) and hemoglobin A1c (percentage %) were presented as the mean±standard deviation among different groups. The missing rates for the FPG and HbA1c were 33.74% and 90.67%, respectively.


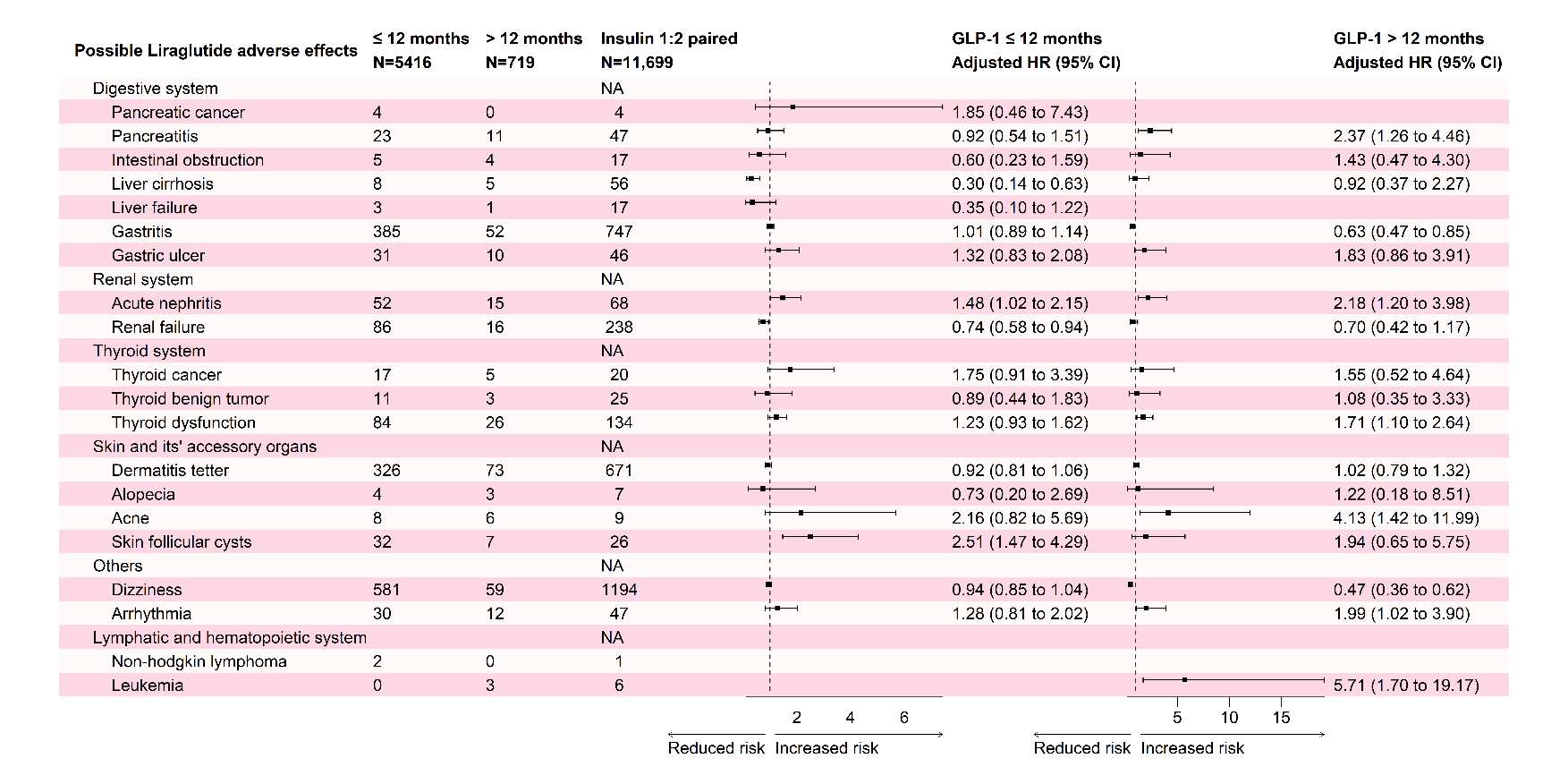
**Figure 3S. Incidence risks of potential adverse effects when Liraglutide compared to the Insulin group (1:2 paired), Liraglutide group stratified by 12 months usage**

Figure 3S presented the potential adverse effects of Liraglutide usage compared with the matched insulin group. After biases-based PSM, various adverse outcomes demonstrated significant association with Liraglutide, including pancreatitis (>12 months), acute nephritis, thyroid dysfunction (>12 months), etc.


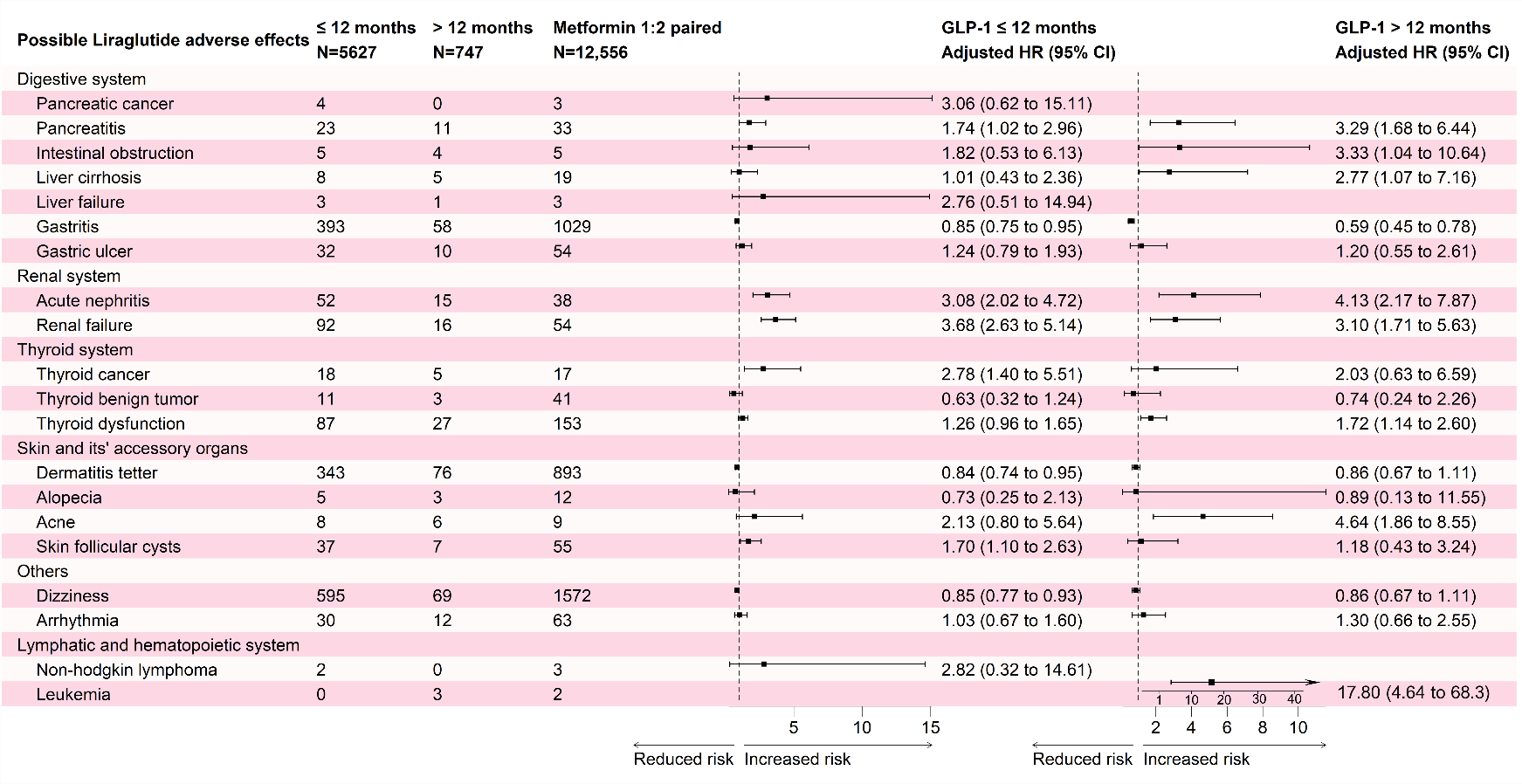
**Figure 4S. Incidence risks of potential adverse effects when Liraglutide compared to the Metformin (1:2 paired), Liraglutide group stratified by 12 months usage**

Figure 4S presented the potential adverse effects of Liraglutide usage compared with the matched Metformin group. After being fully paired by confounding factors, various adverse outcomes demonstrated significant association with Liraglutide, including pancreatitis, intestinal obstruction (>12 months), acute nephritis, renal failure, thyroid cancer (≤12 months), acute nephritis, thyroid cancer (≤12 months), thyroid dysfunction (>12 months), etc.

**Table 5S. Demographic data after PSM among Loxenatide, Insulin, and Metformin groups**

| **Demographic** | **1:2 paired population, n (%)** | |  | **1:2 paired population, n (%)** | |
| --- | --- | --- | --- | --- | --- |
|  | **Loxenatide** | **Insulin (%)** |  | **Loxenatide** | **Metformin (%)** |
|  | **N1=267** | **N2=531** |  | **N1=275** | **N1=550** |
| **Sex, n (%)** |  |  |  |  |  |
| Men | 151 (56.55) | 299 (56.31) |  | 157 (57.09) | 314 (57.09) |
| Women | 116 (43.45) | 232 (43.69) |  | 118 (42.91) | 236 (42.91) |
| Unspecific | 0 (0.00) | 0 (0.00) |  | 0 (0.00) | 0 (0.00) |
| **Age (years), n (%)** |  |  |  |  |  |
| <30 | 11 (4.12) | 21 (3.95) |  | 13 (4.73) | 26 (4.73) |
| 30~ | 144 (54.93) | 288 (54.24) |  | 149 (54.18) | 298 (54.18) |
| 50~ | 85 (31.84) | 168 (31.64) |  | 86 (31.27) | 172 (31.27) |
| ≥70 | 1 (0.37) | 2 (0.38) |  | 1 (0.36) | 2 (0.26) |
| Unspecified | 26 (9.74) | 52 (9.79) |  | 26 (9.45) | 52 (9.45) |
| **Education, n (%)** |  |  |  |  |  |
| High school and below | 136 (50.94) | 269 (50.66) |  | 140 (50.91) | 280 (50.91) |
| Undergraduate and above | 51 (19.10) | 102 (19.21) |  | 55 (20.00) | 110 (20.00) |
| Unspecified | 80 (29.96) | 160 (30.13) |  | 80 (29.09) | 160 (29.09) |
| **Health insurance, n (%)** |  |  |  |  |  |
| Self-pay | 54 (20.22) | 106 (19.96) |  | 54 (19.64) | 108 (19.64) |
| Citizen health insurance | 209 (78.28) | 417 (78.53) |  | 216 (78.55) | 432 (78.55) |
| Other | 4 (1.50) | 8 (1.51) |  | 5 (1.82) | 10 (1.82) |
| **Marriage, n (%)** |  |  |  |  |  |
| Unmarried | 10 (3.75) | 20 (3.77) |  | 10 (3.64) | 20 (3.64) |
| Married | 175 (65.54) | 349 (65.73) |  | 183 (66.55) | 366 (66.55) |
| Divorced or widowed | 2 (0.75) | 2 (0.38) |  | 2 (0.73) | 4 (0.73) |
| Unspecified | 80 (29.96) | 160 (30.13) |  | 80 (29.09) | 160 (29.09) |
| **BMI, n (kg/m^2^, %)** |  |  |  |  |  |
| 18.5~24.9 | 62 (23.22) | 124 (23.35) |  | 63 (22.91) | 126 (22.91) |
| 25.0~29.9 | 102 (38.20) | 204 (38.42) |  | 102 (37.09) | 204 (37.09) |
| 30.0~39.9 | 22 (8.24) | 41 (7.72) |  | 29 (10.55) | 58 (10.55) |
| Unspecified | 81 (30.34) | 162 (30.51) |  | 81 (29.45) | 162 (29.45) |
| **Fasting plasma glucose (mmol/L) *** | 7.38±2.60 | 7.35±2.56 |  | 7.40±2.57 | 7.73±2.39 |
| **Hemoglobin A1c (%) *** | 5.3±4.15 | 6.16±3.16 |  | 5.57±4.01 | 5.85±3.70 |

Note: * The fasting plasma glucose (mmol/L) and hemoglobin A1c (percentage %) were presented as the mean±standard deviation among different groups. The missing rates for the FPG and HbA1c were 29.83% and 88.90%, respectively.


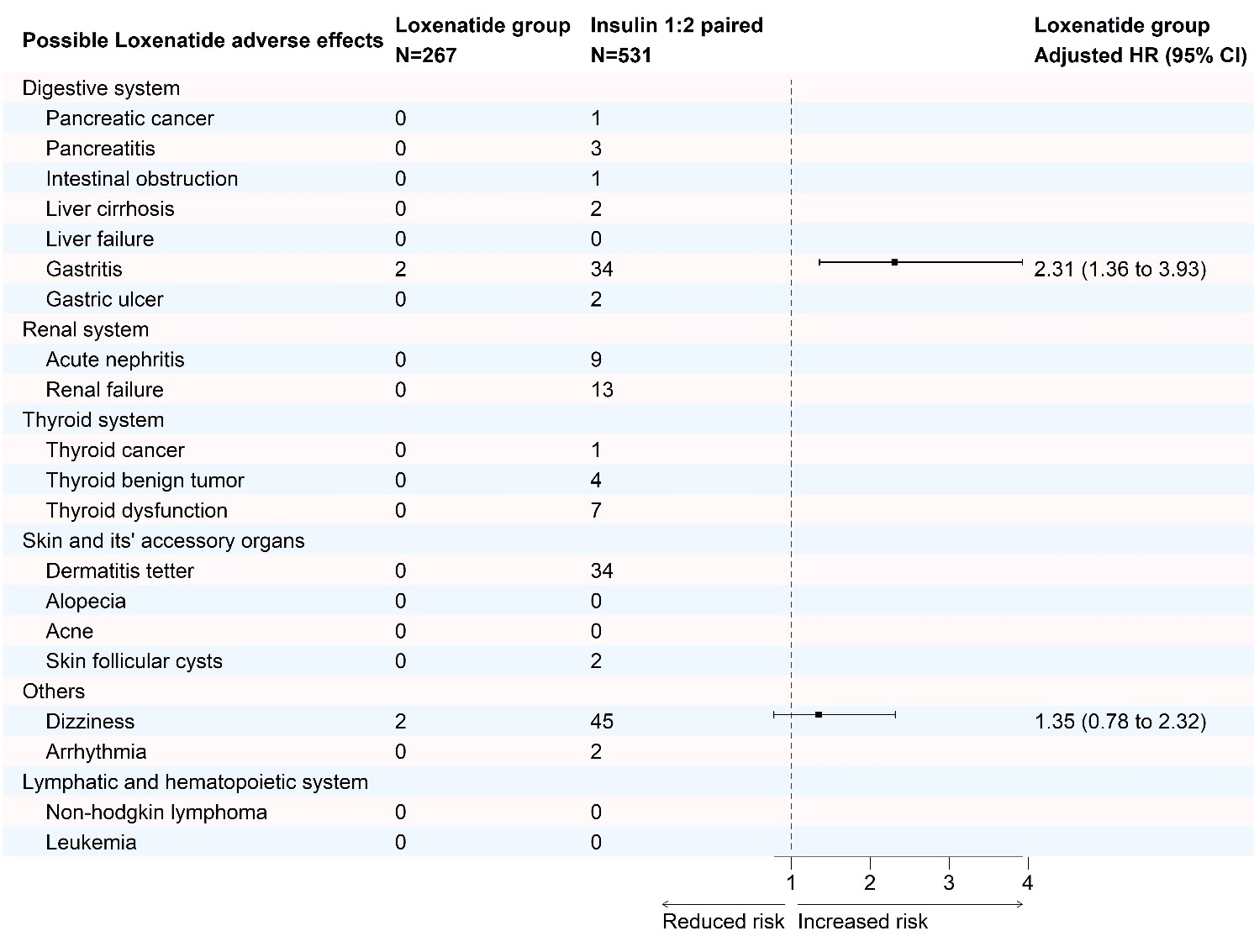
**Figure 5S. Incidence risks of potential adverse effects when Loxenatide group compared to the Insulin group (1:2 paired)**

Due to the relatively limited sample size, there were no significant differences observed in the incidence risks of potential adverse effects associated with Loxenatide compared to the insulin group, except for Gastritis (HR=2.31, 95% CI: 1.36-3.93).


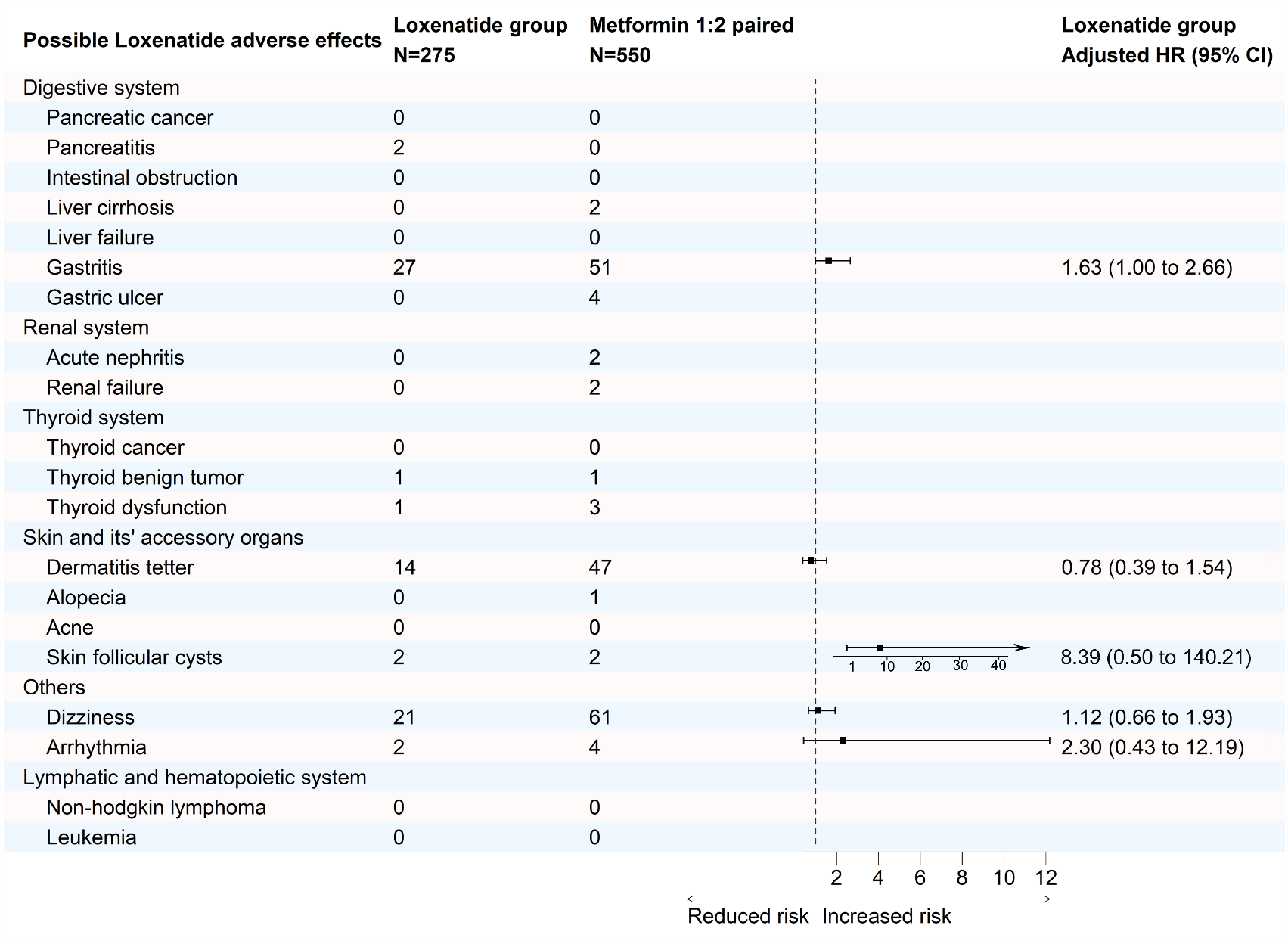
**Figure 6S. Incidence risks of potential adverse effects when Loxenatide group compared to the Metformin group (1:2 paired)**

Similar to the comparison with the insulin group, no significant differences were observed in the incidence risks of potential adverse effects associated with Loxenatide compared to the Metformin group, except for Gastritis (HR=1.63, 95% CI: 1.00-2.66).

**Table 6S. Demographic data after PSM among** **Lixisenatide, Insulin, and Metformin groups**

| **Demographic** | **1:2 paired population, n (%)** | |  | **1:2 paired population, n (%)** | |
| --- | --- | --- | --- | --- | --- |
|  | **Lixisenatide** | **Insulin (%)** |  | **Lixisenatide** | **Metformin (%)** |
|  | **N1=401** | **N2=799** |  | **N1=421** | **N1=841** |
| **Sex, n (%)** |  |  |  |  |  |
| Men | 216 (53.87) | 431 (53.94) |  | 226 (53.68) | 452 (53.75) |
| Women | 185 (46.14) | 368 (46.06) |  | 194 (46.08) | 387 (46.02) |
| Unspecific | 0 (0.00) | 0 (0.00) |  | 1 (0.24) | 2 (0.24) |
| **Age (years), n (%)** |  |  |  |  |  |
| <30 | 4 (1.00) | 8 (1.00) |  | 7 (1.66) | 14 (1.66) |
| 30~ | 138 (34.41) | 273 (34.17) |  | 153 (36.34) | 305 (36.27) |
| 50~ | 154 (38.40) | 308 (38.55) |  | 155 (36.82) | 310 (36.86) |
| ≥70 | 17 (4.24) | 34 (4.26) |  | 18 (4.28) | 36 (4.28) |
| Unspecified | 88 (21.95) | 176 (22.03) |  | 88 (20.90) | 176 (20.93) |
| **Education, n (%)** |  |  |  |  |  |
| High school and below | 170 (42.39) | 337 (42.18) |  | 177 (42.04) | 353 (41.97) |
| Undergraduate and above | 100 (24.94) | 200 (25.03) |  | 113 (26.84) | 226 (26.87) |
| Unspecified | 131 (32.67) | 262 (32.79) |  | 131 (31.12) | 262 (31.15) |
| **Health insurance, n (%)** |  |  |  |  |  |
| Self-pay | 117 (29.18) | 231 (28.91) |  | 118 (28.03) | 235 (27.94) |
| Citizen health insurance | 281 (70.07) | 562 (70.34) |  | 300 (71.26) | 600 (71.34) |
| Other | 3 (0.75) | 6 (0.75) |  | 3 (0.71) | 6 (0.71) |
| **Marriage, n (%)** |  |  |  |  |  |
| Unmarried | 6 (1.50) | 12 (1.50) |  | 11 (2.61) | 22 (2.62) |
| Married | 256 (63.84) | 512 (65.08) |  | 271 (64.27) | 542 (64.45) |
| Divorced or widowed | 8 (2.00) | 13 (1.63) |  | 8 (1.90) | 15 (1.78) |
| Unspecified | 131 (32.67) | 262 (32.79) |  | 131 (31.12) | 262 (31.15) |
| **BMI, n (kg/m^2^, %)** |  |  |  |  |  |
| 18.5~24.9 | 105 (26.18) | 208 (26.03) |  | 105 (24.94) | 209 (31.15) |
| 25.0~29.9 | 128 (31.92) | 255 (31.91) |  | 130 (30.88) | 260 (30.92) |
| 30.0~39.9 | 37 (9.23) | 74 (9.26) |  | 55 (13.06) | 110 (13.08) |
| Unspecified | 131 (32.67) | 262 (32.79) |  | 131 (31.12) | 262 (31.15) |
| **Fasting plasma glucose (mmol/L)*** | 7.38±2.60 | 7.35±2.56 |  | 7.55±2.51 | 7.60±2.47 |
| **Hemoglobin A1c (%) *** | 6.85±2.85 | 6.16±3.16 |  | 6.82±2.33 | 6.76±3.44 |

Note: * The fasting plasma glucose (mmol/L) and hemoglobin A1c (percentage %) were presented as the mean±standard deviation among different groups. The missing rates for the FPG and HbA1c were 28.72% and 89.59%, respectively.


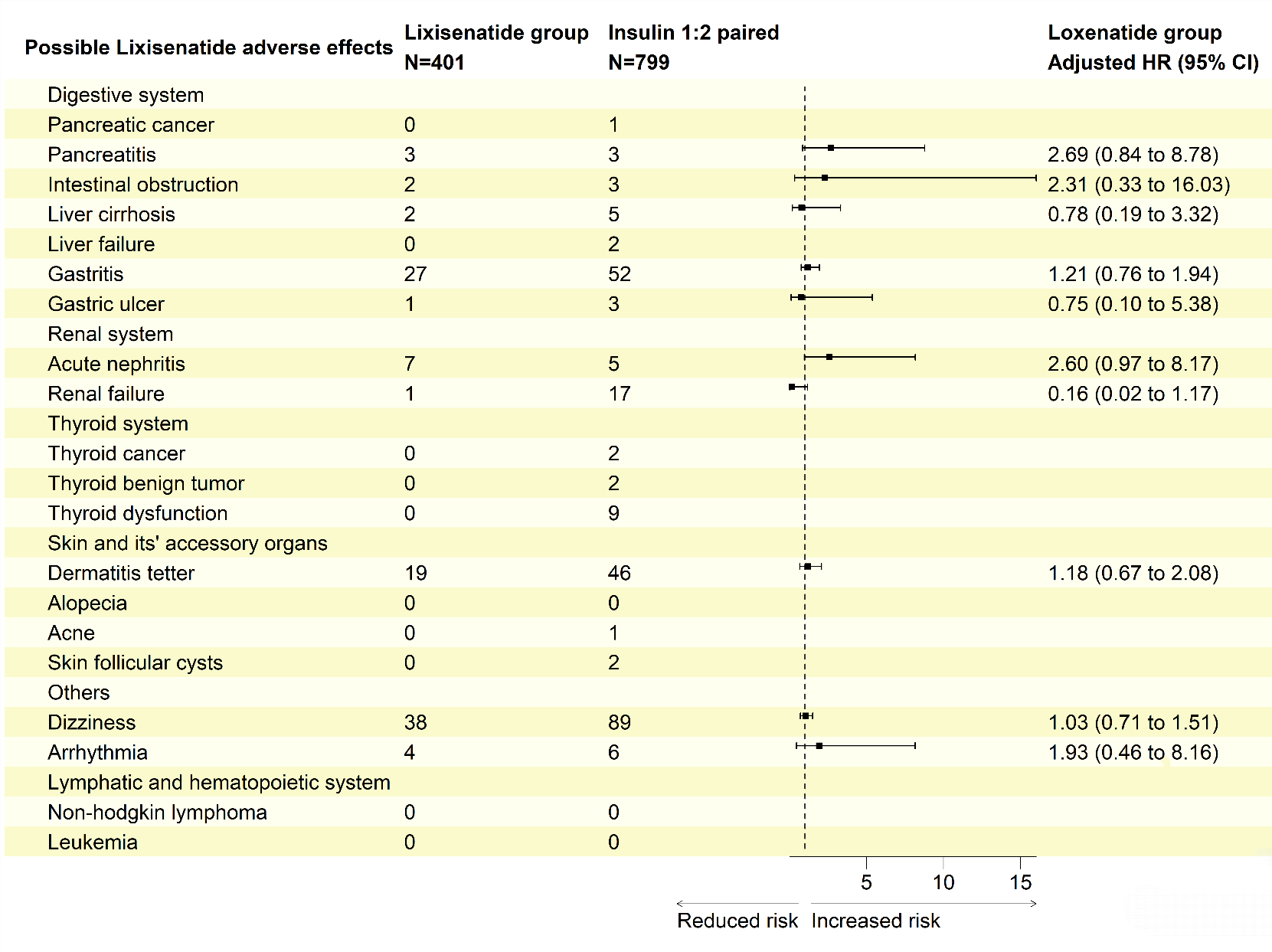
**Figure 7S. Incidence risks of potential adverse effects of Lixisenatide in comparison to the Insulin group (1:2 paired).**

Due to the relatively limited sample size, there was a non-substantial increase in the incidence risk of potential adverse effects associated with Lixisenatide compared to the insulin group. However, incidence risks of pancreatitis (HR:2.69, 95%CI: 0.84-8.78) and acute nephritis (HR: 2.60, 95%CI: 0.97-8.17) were observed near the borderline of significance.


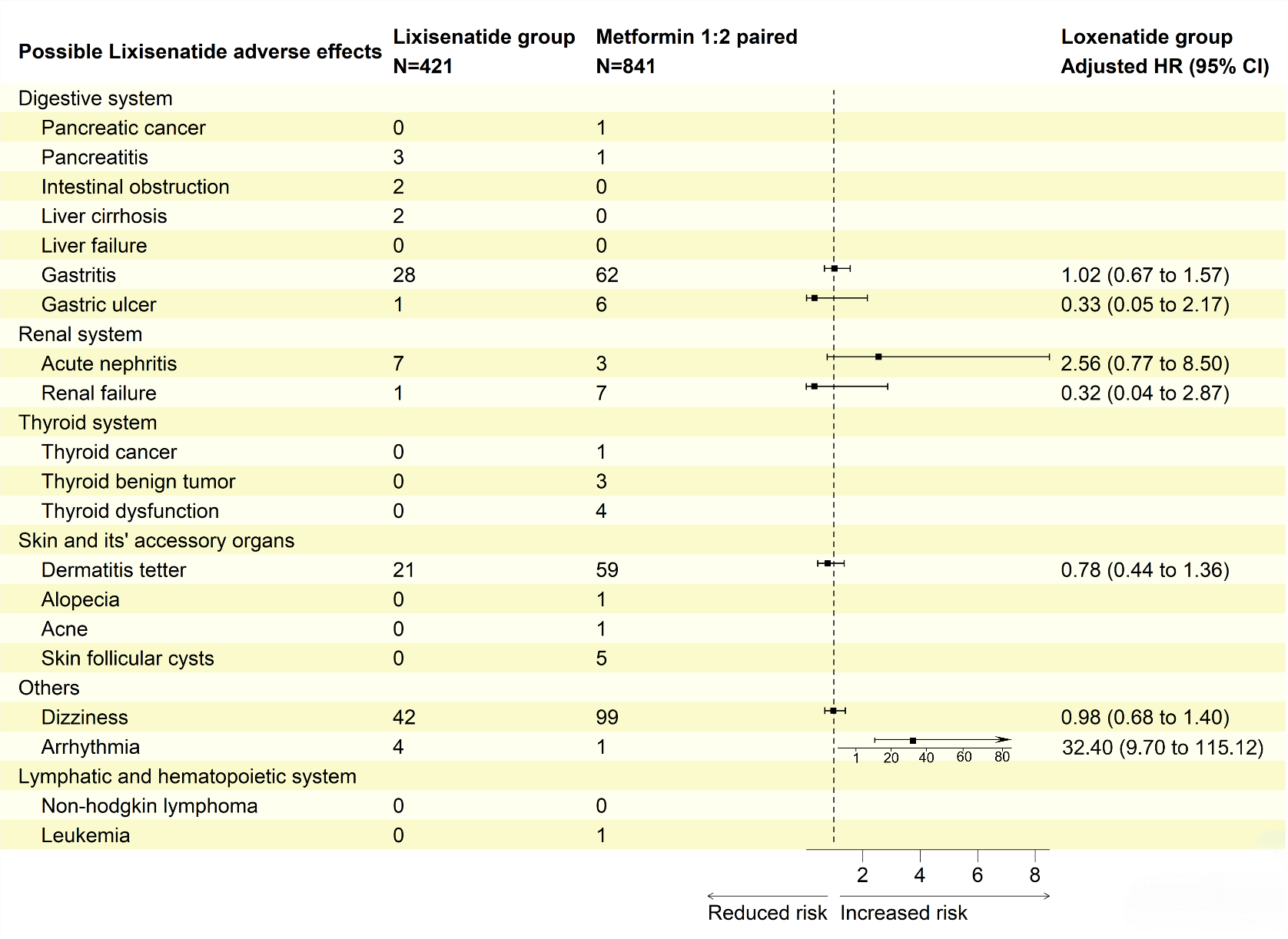
**Figure 8S. Incidence risks of potential adverse effects of Lixisenatide in comparison to the Metformin group (1:2 paired).**

Similar to supplemental figure 6S, non-substantial increased incidence risk of potential adverse effects associated with Lixisenatide compared to the Metformin group. However, the incidence risk of acute nephritis (HR: 2.56, 95%CI: 0.77-8.50) was reached the significance borderline.

**Table 7S. Demographic data after PSM among** **Semaglutide, Insulin, and Metformin groups**

| **Demographic** | **1:2 paired population, n (%)** | |  | **1:2 paired population, n (%)** | |
| --- | --- | --- | --- | --- | --- |
|  | **Semaglutide** | **Insulin (%)** |  | **Semaglutide** | **Metformin (%)** |
|  | **N1=726** | **N2=1,451** |  | **N1=751** | **N1=1,499** |
| **Sex, n (%)** |  |  |  |  |  |
| Men | 429 (59.09) | 857 (40.94) |  | 311 (41.41) | 620 (41.36) |
| Women | 297 (40.91) | 594 (40.94) |  | 440 (58.59) | 879 (58.64) |
| Unspecific | 0 (0.00) | 0 (0.00) |  | 0 (0.00) | 0 (0.00) |
| **Age (years), n (%)** |  |  |  |  |  |
| <30 | 36 (4.96) | 72 (4.96) |  | 42 (5.59) | 82 (5.47) |
| 30~ | 326 (44.90) | 652 (44.93) |  | 344 (45.81) | 687 (45.83) |
| 50~ | 227 (31.27) | 454 (31.29) |  | 228 (30.36) | 456 (30.42) |
| ≥70 | 23 (3.17) | 45 (3.10) |  | 23 (3.06) | 46 (3.07) |
| Unspecified | 114 (15.70) | 228 (15.71) |  | 114 (15.18) | 228 (15.21) |
| **Education, n (%)** |  |  |  |  |  |
| High school and below | 249 (32.30) | 497 (34.25) |  | 254 (33.82) | 508 (33.89) |
| Undergraduate and above | 180 (24.79) | 360 (24.81) |  | 201 (26.76) | 399 (26.62) |
| Unspecified | 297 (40.91) | 594 (40.94) |  | 296 (39.41) | 592 (39.49) |
| **Health insurance, n (%)** |  |  |  |  |  |
| Self-pay | 173 (23.83) | 346 (23.85) |  | 175 (23.30) | 349 (23.28) |
| Citizen health insurance | 545 (75.07) | 1089 (75.05) |  | 568 (75.63) | 1134 (75.65) |
| Other | 8 (1.10) | 16 (1.10) |  | 8 (1.07) | 16 (1.07) |
| **Marriage, n (%)** |  |  |  |  |  |
| Unmarried | 17 (2.34) | 34 (2.34) |  | 23 (3.06) | 45 (3.00) |
| Married | 406 (55.92) | 812 (55.96) |  | 425 (56.59) | 848 (45.57) |
| Divorced or widowed | 6 (0.83) | 11 (0.76) |  | 7 (0.93) | 14 (0.93) |
| Unspecified | 297 (40.91) | 594 (40.94) |  | 296 (30.41) | 592 (39.49) |
| **BMI , n (kg/m^2^, %)** |  |  |  |  |  |
| 18.5~24.9 | 190 (26.17) | 379 (26.12) |  | 190 (25.30) | 380 (25.35) |
| 25.0~29.9 | 170 (23.42) | 340 (23.43) |  | 172 (22.90) | 343 (22.88) |
| 30.0~39.9 | 69 (9.50) | 138 (9.51) |  | 93 (12.38) | 184 (12.27) |
| Unspecified | 297 (40.91) | 594 (40.94) |  | 296 (39.41) | 592 (39.49) |
| **Fasting plasma glucose (mmol/L)*** | 7.28±2.41 | 7.45±2.71 |  | 7.24±2.36 | 7.42±2.34 |
| **Hemoglobin A1c (%) *** | 6.57±3.09 | 6.37±2.97 |  | 6.57±3.09 | 6.41±3.34 |

Note: * The fasting plasma glucose (mmol/L) and hemoglobin A1c (percentage %) were presented as the mean±standard deviation among different groups. The missing rates for the FPG and HbA1c were 30.18% and 90.61%, respectively.


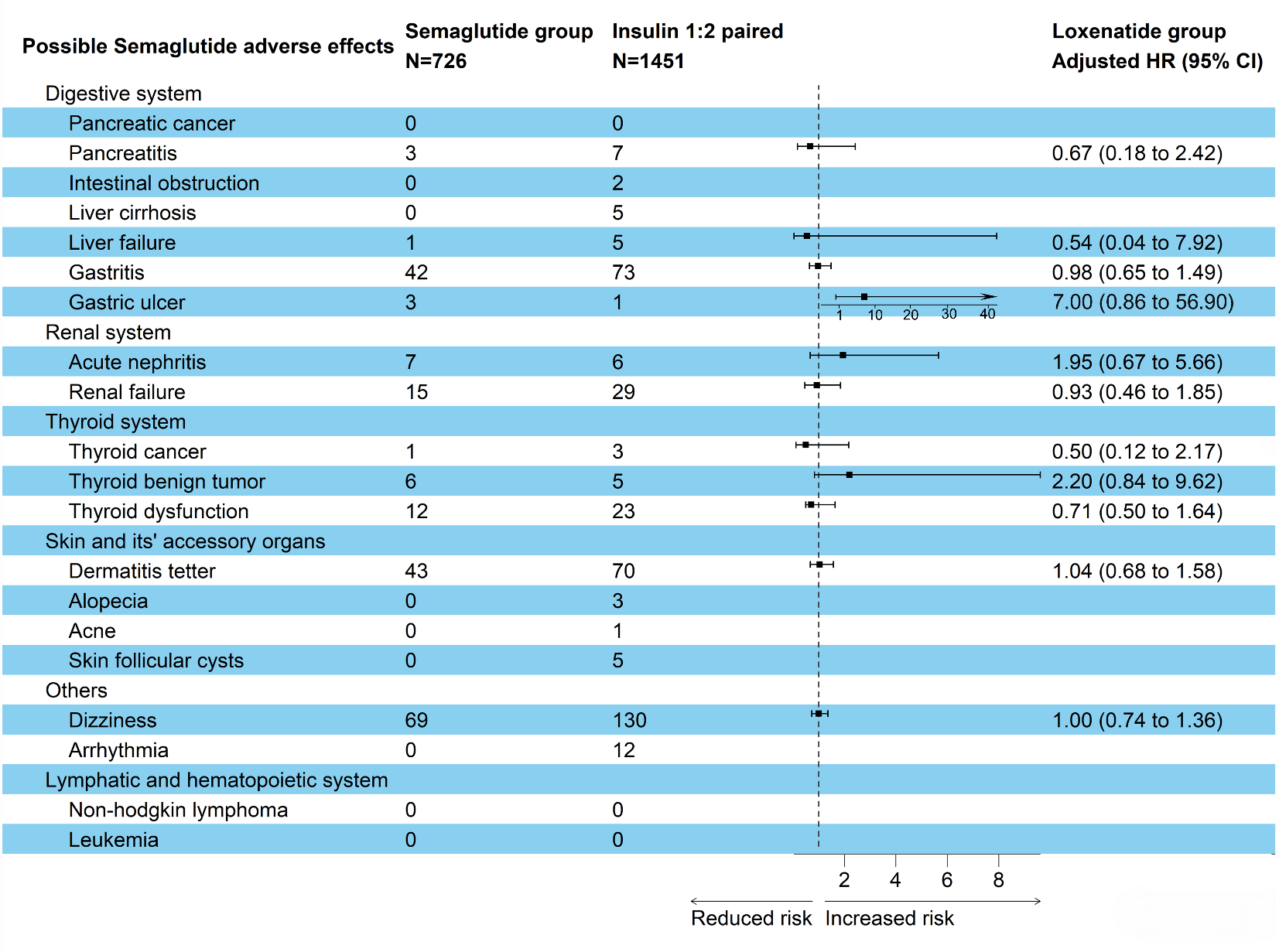
**Figure 9S. Incidence risks of potential adverse effects when Semaglutide group compared to the Insulin group (1:2 paired).**

When compared to the biased-matched insulin group, non-significant increased incidence risk of potential adverse effects associated with Semaglutide was observed when compared to the the insulin group. Of note, the incidence risks of thyroid benign tumor (HR:2.20, 95%CI: 0.84-9.62) were observed reaching borderline.


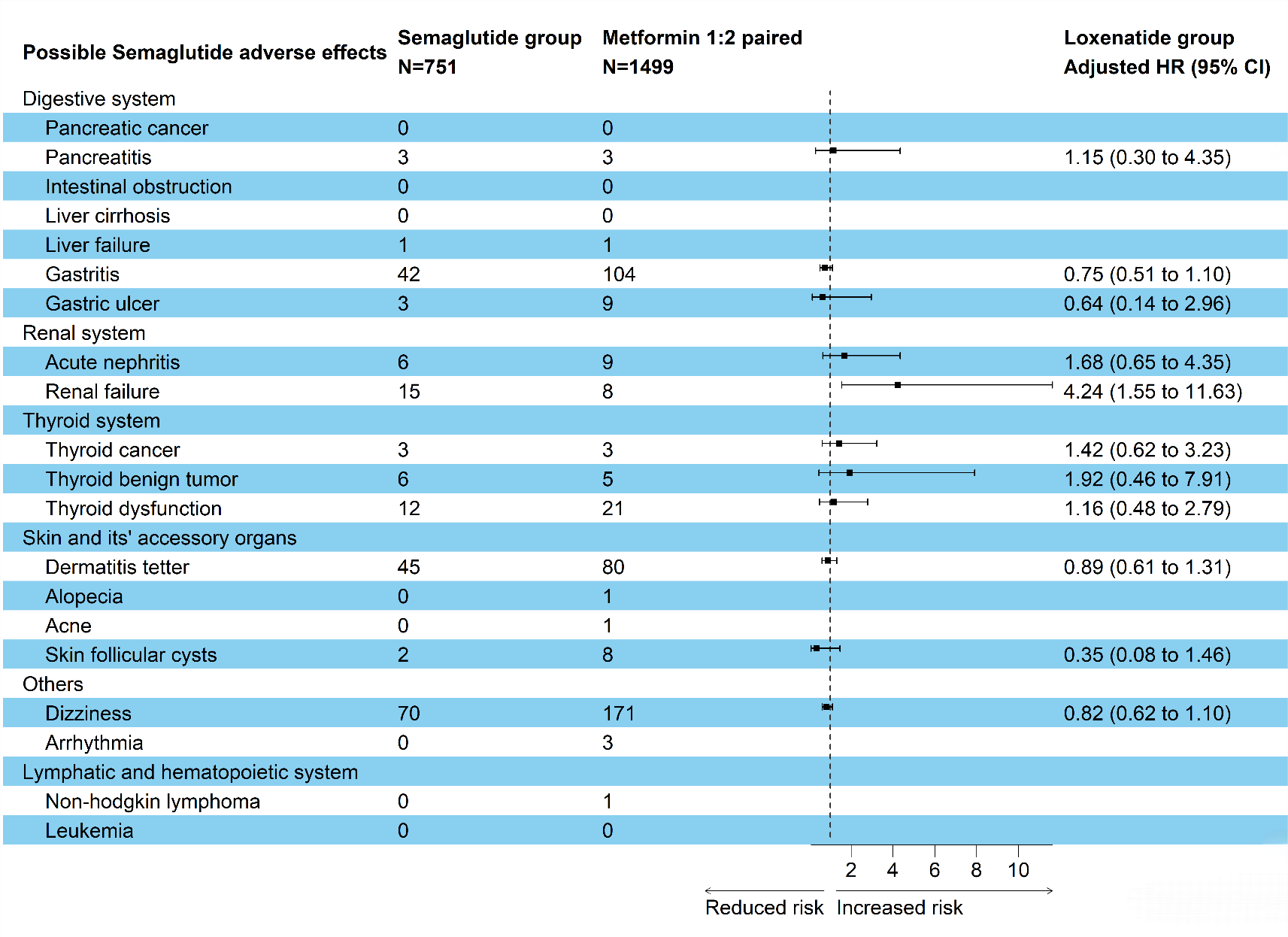
**Figure 10S. Incidence risks of potential adverse effects when Semaglutide group compared to the Metformin group (1:2 paired).**

When compared to the biased-matched Metformin group, significantly increased incidence risk of renal failure (HR:4.24, 95%CI: 1.55-11.63) was found.
